# Hospital Meetings, &c.

**Published:** 1900-05-12

**Authors:** 


### HOSPITAL MEETINGS, &C.

### KING'S COLLEGE HOSPITAL.

Annual Festival Dinner.

The annual festival dinner of the King's College Hospital was held at the Hotel Metropole on Monday evening, May 7th, under the presidency of the Hon. W. F. D. Smith, M.P. There was a large company, including the Viscount Dillon (chairman of the Committee of Management), Mr. Charles Awdry (treasurer), Sir Alfred C. Lyall, G.C.I.E., K.C.B., Sir W. Robinson, G.C.M.G., Sir Charles J. Lyall, K.C.S.I., C.I.E., the Archdeacon of London, and many others.

The usual loyal sentiments having been expressed,

The Chairman, on rising to give the toast of the evening, "Success to King's College Hospital," said the toast was not a mere barren form of words, for he felt sure that not only those specially connected with the hospital, but everyone present, would, by their acts, do what they could to make the institution a real success this year. He was treasurer of a kindred institution, and he knew that the person occupying that position had not only to administer the funds which he might have at his disposal, but also, very frequently, to ask for the funds to administer. He had been in the position of a beggar more than once, and he was therefore able to sympathise with other beggars—(laughter)—and more especially with his friend Mr. Awdry, the treasurer of King's College Hospital. He thought it would be agreed by all, however, that Mr. Awdry merited little sympathy, for his cause was good, and therefore his funds should be easily obtained. He (the chairman) had no doubt whatever as to the goodness of the cause which he had to plead on the present occasion. The only doubt in his mind was that he would not be able to do it sufficient justice; but he could at any rate say, with perfect confidence, that he had never felt a more genuine interest in any institution than he had in the institution for the benefit of which they had met that evening. (Applause.) He had had an opportunity in the course of last week of visiting the hospital in company with the treasurer, the warden, and the sister-matron, and he could honestly say that the motherly pride which the sister-matron showed over everything was amply justified by all he saw—(applause)—for it seemed to him there was a general atmosphere of comfort and life in all the wards into which he went that was calculated to relieve the somewhat sad impressions which came, perhaps, upon those who were not as accustomed as were many of those now present to the somewhat saddening atmosphere of a hospital. It was quite evident to him that everything possible was done to make the lot of the 2,500 patients who annually found rest and comfort within the walls of the hospital as pleasant as anything under such circumstances could be. But a work of this magnitude could not be done for nothing, and it was a serious matter for any hospital committee to have to raise a sum of nearly £19,000 a year to carry on its work efficiently, and this was especially the case with the committee of King's College Hospital, because the committee had had to find an extra £3,000 this year for reflooring and relighting the wards—£3,000 which had evidently been well spent, for both the reflooring and the relighting had added enormously to the comfort and utility of those wards in which the improvements had been carried out. One of the things he had to ask his hearers to do that night was to help the committee to pay off the debt that still remained upon that capital expenditure, amounting to something over £1,000. King's College Hospital was, perhaps, not more unfortunate than other hospitals in having to find a large amount of money yearly beyond what it could with certainty lay its hand upon, which was extremely limited. From the funds arising out of invested money, and from annual subscriptions, which, of course, came with more certainty than donations and legacies—they had a fund of some £5,000 towards meeting the annual expenditure, but if they subtracted that sum from the £19,000 which the committee annually expended, they would see there was still a very large sum indeed for which the hospital had to depend entirely upon the liberality of friends. If he might judge from the suggestion which was made to him by both the sister-matron and the treasurer, there were improvements which, if they had the funds, the committee would very readily undertake. In the first place, there was undoubtedly room for improvement in the out-patient and casualty departments. (Hear, hear.) He visited both those departments, and there was no doubt in his mind that they were not big enough, convenient enough, or light enough to satisfactorily deal with the 22,000 patients who came during the course of the year to have their hurts and maladies attended to. Moreover, if it were possible for the committee to deal with those two departments as they would like to, there would be considerable space set free for them to make arrangements for wards which at the present time were sorely needed—wards to deal with cases which required separate treatment, such as diseases of the ear and throat, which now had not sufficient accommodation, and which would benefit enormously if such accommodation could be provided. Then the committee would like to provide, if possible, better accommodation for the nurses when off duty. There was no body who better deserved all the consideration they could give them than those who spent their lives and energies in nursing the sick poor in our great hospitals. (Applause.) He was well aware that we were passing through a year of great stress and strain; a time when people had to divert their thoughts from ordinary courses to sons, brothers, or fathers fighting in South Africa; a time when unaccustomed demands were made for our fellow-men in the East, our soldiers in the South, and those who had suffered by fire in the West. New inroads were being made day by day upon our charity, but was it not for that reason that there was the more occasion for our help, so that when peace should take the place of war, and plenty reign instead of famine, we might be able to look back on this year and say with truth that our old institutions had not suffered, that the stream of charity had not been diverted, but rather that it had overflowed its banks in such volume that it had been sufficient to fill channels old and new alike (Loud applause.) It was with the conviction that those present, as far as in them lay, would make that great effort that he gave with every confidence the toast of "Success to King's College Hospital."

The Viscount Dillon (chairman of the Committee of Management), in reply, said that in the last 20 years the hospital had, in a way, changed its conditions, the neighbourhood having considerably altered for the better; but, as of old, they still had the poor always with them. The large market close by supplied a considerable number of casualty cases for treatment in their wards, and patients came to the hospital from all parts of the country. Referring to the war, his Lordship said it was very near to them, for not only had all of them friends or relatives who were engaged in South Africa, but it was interesting also to know that the hospital had taken its own part in the matter. In Professor Cheyne, Mr. Cheatle, and Mr. Cargill it had supplied three of the most efficient officers to assist the regular military authorities in mitigating the troubles of the soldiers. They had also lent—he hoped only for a short time—one of their sisters. Not having sufficient confidence in them, apparently, she resigned before going out; but he was not alone in saying that they would be very glad if, when she came back, they could see their way to obtaining her very efficient services again for the hospital. (Applause.) With regard to the improvements to which the chairman had referred, the relighting had been very considerably assisted by the private munificence of various gentlemen who did not wish their names to be mentioned; but the cost of the reflooring has been a direct charge on the hospital, and funds were needed to meet this expense. It had been said that the hospital was "like home," but he thought it would be a great abuse of public charity if the poor people who came to the hospital found such a state of things there as existed in their own homes. Anyone who visited the hospital either from this country or any other would, he thought, be prepared to admit that the greatest possible amount of comfort compatible with the strict management which was necessary in a hospital was found there. There were doubtless many things which people unacquainted with hospitals might not see, and might wish to see, but it must be remembered that hospital life was an abnormal state of life. As in previous years, the hospital had earned the approbation of the committee of the Prince of Wales's Fund, the members of which came, and, having made a most searching examination into all the arrangements, declared themselves well satisfied. Of this satisfaction they gave ample proof by making a grant of £1,000, and a further grant in earnest of the improvement which they hoped some day to make. The schemes for spending any magnificent sums which they might receive were very numerous. (Laughter.) There would be a thousand ways of spending the money, and 999 of them were good ways, but it would resolve itself into one when it came to the point. They were waiting for means to do a great deal, and those who were acquainted with the hospital would know that not only the nurses' leisure room was utterly inadequate for the purposes to which it was assigned at present, but the accommodation for their living was not what the committee would wish it to be, and although they could not hope to get more work from the nurses, who already did their utmost, they would have the satisfaction of feeling that the nurses were doing their work more easily and with less interference with their own comfort and health. Concluding, his lordship said that all interested in the institution were endeavouring in every way to maintain the good reputation and the best of the past traditions of King's College Hospital. (Applause.)

Mr. A. H. A. Morton, M.P., gave "The National Services: The Church, the Law, and the Naval and Military

Forces," which was responded to by Archdeacon Sinclair, Mr. C. P. Johnson, and Major Ferguson.

The Rev. Dr. Robertson, in proposing "The Medical Staff of the Hospital," referred to the staff as "the man behind the gun," and declared the hospital to be "well armed at all points in the battle against disease."

This toast was acknowledged by Dr. Ferrier, the senior physician of the hospital, and the health of the chairman was also drunk.

During the evening it was announced by the Treasurer, amid considerable applause, that the total proceeds of the festival were £2,765 5s., including £200 from the chairman of the dinner, £500 from Mr. W. H. Bell, £100 from Mr. C. Peace Serocold, £52 10s. from Mr. Conrad Wilkinson, £50 from the Duke of Bedford, £52 10s. from Mr. Frederick Lee, £525 from Messrs. W. H. Smith and Sons, £105 from Mr. C. P. Johnson, and £50 from Mr. C. Awdry.

### METROPOLITAN HOSPITAL.

The sixty-fourth annual meeting of the Metropolitan Hospital was held on Monday, 7th inst., Mr. C. J. Thomas, the chairman, presiding. The accounts presented showed ordinary receipts £7,272, and legacies £2,704. The ordinary expenditure was £9,390, and the extraordinary expenditure on repairs and building improvements £270. The report pointed out that, though the total receipts for the year were less than those of 1898 by £2,629, the difference was mainly in the legacies, which were £2,413 less; while in 1898 the hospital had received £630 from the Shoreditch Guardians. The total expenditure of £9,660 compared with £10,081 in 1898. Special attention was called to the fact that the Prince of Wales's Fund had increased their grant from £500 to £1,000, and the Chairman reminded the meeting that these grants were not sent in ignorance, but that each year they received a deputation from the Fund who thoroughly inspected every detail of the hospital, making such suggestions as occurred to them for improvement or economy. The festival dinner held on April 24th, 1899, at which Mr. H. L. Webster Lawson presided, realised £2,841. Mr. Lawson had become a vice-patron of the hospital. During 1899 the in-patients numbered 867; the out-patients 31,245, with attendances of 101,597. The average stay of each in-patient was 29½ days—the same as in the previous year. The daily average number of beds occupied was 70. There were 4,179 members of the provident department; the total attendances were 26,384, and 7,868 home visits were made by the medical officers of the department. During the autumn, at the invitation of the Metropolitan Asylums Board, the committee had placed at their disposal, in connection with the serious outbreak of enteric, two empty wards with accommodation for 24 and 12 beds. The Board sent the necessary furniture and bedding, and agreed to pay a sum to cover the cost. These wards were occupied from November 14th to February 21st. Great regret was expressed at the death of Mr. Walter J. Fry, who joined the committee on the death of their late chairman, his father, Mr. Joseph Fry, in 1896. His brother, Mr. F. W. Fry, had been elected to take his place. Regret was felt at the resignation, owing to pressure of business, of Mr. John Carter, who had been a member of the committee for nearly eight years. The resignations were also reported of Dr. Gow (for ten years obstetric physician), Dr. A. G. Phear, and Mr. J. S. Sloane. Dr. H. Playfair, Dr. W. Langdon Brown, and Mr. P. L. Daniel had been appointed to fill these vacancies. Miss Ethel Barwell, the sister of the children's ward, had resigned to take up the duties of matron of the Belgrave Hospital; Miss Cosgrove, the sister of the women's ward, had resigned to get married; and Miss Leonard and Miss Lindsey had been appointed to these vacancies. Miss Presland, the staff nurse of the Jewish ward, had been promoted to be sister of that ward. Mention was made of the kindness of the Hackney Vestry and the North Metropolitan Tramways Company in paving the roadway in front of the hospital with wood—an improvement greatly appreciated by the staffs of the hospital as well as by the patients. Thanks were recorded to Mr. Neve for his gift of a horse, and to the Rev. Ernest Sanders for a pony carriage for the use of the patients at the Convalescent Home, as well as to Miss Baring, who had placed two cots at her Home at High Beech, Epping Forest, at the disposal of convalescent children, patients at the hospital. At a meeting held at Surrey House in July, a Ladies' Association had been formed, and the committee were confident that it would be of the utmost advantage in gathering fresh support. The Princess Louise had become president, with Lady Battersea and Mrs. Ernest Sanders as treasurers; the Rev. Ernest Sanders (to whose efforts the formation of the association was due) being elected hon. secretary. The membership had steadily increased, and was now 153. One hundred guineas had been given to the general funds and fifty guineas to defray the cost of laying out the grounds at the Convalescent Home. The association was at the moment engaged in promoting a grand afternoon concert at the Queen's Hall for the 22nd inst.

After the adoption of the report and accounts a resolution was carried substituting the title of Surgeon for Diseases of Women for that of Obstetric Physician. This, the Chairman said, was recommended by the medical staff as more correctly describing the position held, and as being more comprehensive and specially applicable to the post.

The re-election of the retiring members of the committee, Mr. L. Davidson, Mr. W. G. Jeffreys, and Lieut.-Colonel Montefiore, with the addition of Mr. S. Gurney Shephard, jun., and Mr. A. Lister, was then voted, and the proceedings ended with a very warmly recommended vote of thanks to the chairman.

